# Impact of single-dose HPV vaccination on HPV 16 and 18 prevalence in South African adolescent girls with and without HIV

**DOI:** 10.1093/jncimonographs/lgae041

**Published:** 2024-11-12

**Authors:** Sinead Delany-Moretlwe, Dorothy A Machalek, Danielle Travill, Kathy Petoumenos, Dorothy C Nyemba, Zizipho Z A Mbulawa, Nontokozo Ndlovu, John M Kaldor, Helen Rees

**Affiliations:** Wits RHI, University of the Witwatersrand, Johannesburg, South Africa; Kirby Institute, University of New South Wales, Sydney, Australia; Centre for Women’s Infectious Diseases, The Royal Women’s Hospital, Melbourne, Australia; Wits RHI, University of the Witwatersrand, Johannesburg, South Africa; Kirby Institute, University of New South Wales, Sydney, Australia; Wits RHI, University of the Witwatersrand, Johannesburg, South Africa; National Health Laboratory Service, Nelson Mandela Academic Hospital, Mthatha, South Africa; Wits RHI, University of the Witwatersrand, Johannesburg, South Africa; Kirby Institute, University of New South Wales, Sydney, Australia; Wits RHI, University of the Witwatersrand, Johannesburg, South Africa

## Abstract

**Background:**

The World Health Organization has endorsed single-dose human papillomavirus (HPV) vaccination, but data on the impact on HPV prevalence in high HIV burden settings are limited.

**Methods:**

A single-dose bivalent HPV vaccine was delivered to adolescent girls in grade 10 in a schools-based campaign in 1 district in South Africa. Impact on HPV 16 and 18 prevalence was evaluated using repeat cross-sectional surveys. A clinic-based survey in girls aged 17-18 years established HPV 16 and 18 prevalence in a prevaccine population (n = 506, including 157 living with HIV) in 2019 and was repeated in the same age group and sites in a single-dose eligible population in 2021 (n = 892, including 117 with HIV). HPV DNA was detected on self-collected vaginal swabs using the Seegene Anyplex II HPV 28. Population impact was estimated overall and by HIV status using prevalence ratios adjusted for differences in sexual behavior between surveys.

**Results:**

Single-dose vaccination campaign coverage was 72% (4807 of 6673) of eligible girls attending high school (n = 66) in the district. HPV 16 and 18 prevalence was 35% lower in the postvaccine survey overall (adjusted prevalence ratio = 0.65, 95% confidence interval [CI] = 0.51 to 0.83; *P* < .001) and 37% lower in those living with HIV (adjusted prevalence ratio = 0.63, 95% CI = 0.41 to 0.95; *P*  = .026). No protective effect was seen for nonvaccine oncogenic HPV types 33, 35, 39, 51, 52, 56, 58, 59, or 68 overall (adjusted prevalence ratio = 1.14, 95% CI = 1.03 to 1.26; *P* = .011) or in those living with HIV (adjusted prevalence ratio = 1.00, 95% CI = 0.83 to 1.21. *P* = 0.99).

**Conclusion:**

These data provide reassuring evidence of single-dose impact on population-level HPV 16 and 18 prevalence in a South African population, irrespective of HIV status.

Since initial licensure in 2006, prophylactic vaccines against human papillomavirus (HPV) have been introduced into more than 137 countries worldwide (1). Currently, 6 prophylactic vaccines are licensed. All are highly effective against high-risk HPV types 16 and 18 that are responsible for approximately 70% of cervical cancers globally (2). The quadrivalent and nonavalent vaccines also prevent low-risk HPV types 6 and 11 associated with anogenital warts. The nonavalent vaccine provides protection against 5 additional high-risk HPV types: HPV 31, 33, 45, 52, and 58. The bivalent and quadrivalent vaccines reportedly provide partial but short-lived cross-protection against HPV 31 and 45 and HPV 31, respectively (3).

More than a decade after HPV vaccine implementation, a meta-analysis involving more than 60 million individuals in high-income countries comparing pre- and postvaccination periods showed an 83% decline in HPV 16 and 18 prevalence in girls aged 13-19 years 5 years after vaccination compared with prevaccination prevalence in this age group (4). These reductions in infection have translated into impacts on HPV-associated disease, with statistically significant reductions in anogenital warts, precancerous cervical lesions, and cervical cancer observed (4,5). Despite these considerable impacts, progress on HPV vaccine introduction into low- and middle-income countries (LMIC), where more than 90% of cervical cancer deaths occur, has been slow; by 2019, only 41% of LMIC had included HPV vaccines in their immunization schedule (6,7). In 2020, the World Health Organization (WHO) recommended that 90% of all girls be vaccinated against HPV by age 15 years by 2030, as part of efforts to accelerate the global elimination of cervical cancer (8).

HPV vaccine introduction in LMIC has been hampered by cost, program complexity, supply chain constraints, low awareness of vaccine impact, and vaccine hesitancy (9). Reducing the number of doses in the vaccination schedule could potentially increase program efficiency, reduce cost, or allow coverage of a wider age band, leading to greater global vaccine coverage. Post hoc analyses from a large trial in Costa Rica provided initial evidence of the potential for a single dose of HPV vaccine to provide a satisfactory immune response and equivalent protection against incident HPV 16 and 18 that was sustained over time compared with 2 or 3 vaccine doses (10). These initial findings have subsequently been confirmed in postlicensure observational studies and, most recently, in a prospective randomized controlled trial in Kenya that demonstrated 98%-99% single-dose vaccine efficacy against vaccine type-specific persistent HPV out to 3 years (11,12) In response, WHO recommended an alternative, off-label single-dose vaccine schedule for use in girls and boys aged 9-20 years in November 2022 (13). There was insufficient evidence however to make a recommendation around single-dose vaccination in people living with HIV, given limited vaccine efficacy studies in this population (14). This has led to concerns about adopting a single-dose policy in high HIV prevalence settings like South Africa, where women living with HIV have a sixfold higher risk of cervical cancer and require adequate protection against high-risk HPV infection (15).

South Africa was one of the first African countries to launch a national schools-based HPV vaccination program in 2014. Two doses of the bivalent vaccine (Cervarix) are offered to girls who are in grade 4 in primary school and 9 years and older, in a grade-based campaign that is run at schools in March and September annually. An early evaluation of the program showed 87% first-dose program coverage, but subsequent program coverage rates have been lower with second-dose program coverage less than 56% over the past 5 years (16,17). To guide further decision making, we evaluated the population impact of a single-dose vaccine schedule on HPV prevalence overall and by HIV status, when delivered as a catch-up campaign to adolescent girls in grade 10 (aged 15-16 years) in a single district in the Free State, South Africa.

## Methods

### Single-dose vaccination campaign

Detailed methods have been described elsewhere (18). Briefly, we offered a single dose of the bivalent HPV vaccine (Cervarix) to all adolescent girls in grade 10 (expected age 15-16 years) attending 66 high schools in 1 district in South Africa from February to May 2019. A total of 6673 girls in grade 10 were enumerated using Department of Education records, in a district with more than one-half million inhabitants. The single-dose campaign was modeled on the national HPV vaccination program and implemented by a dedicated study team that provided all resources including vaccines, refrigerators, staff, and transport. The Department of Health required written parental or guardian consent and learner assent. Prior to vaccine administration, potential recipients were assessed for vaccine eligibility; those without written consent, pregnant or breastfeeding, or with an acute illness in the previous 30 days were not vaccinated. Basic demographic and vaccine information was captured in an electronic vaccine register held by the study team.

### Study design and population

Single-dose vaccine impact on population HPV prevalence was assessed in repeat cross-sectional surveys in adolescent girls aged 17-18 years attending public sector primary health-care clinics, prior and subsequent to the single-dose campaign. The prevaccine survey in 2019 recruited girls aged 17-18 years who were therefore above the target age for vaccination in 2014 when the program started in South Africa. The postvaccine survey in 2021 recruited girls aged 17-18 years who would have been eligible for and exposed to the school-based single-dose HPV campaign conducted in the district in 2019. Participants were recruited from the same clinic sites in the district in both surveys. In South Africa, primary health care that is both curative and preventive is delivered through public sector clinics, available within 5 km to more than 90% of the population and free at the point of use (19,20). Adolescents routinely access these services for contraception, HIV testing and treatment, and management of sexually transmitted infections (STIs) and other common ailments.

The repeat clinic-based cross-sectional survey design is commonly used as a strategy for assessing population-level vaccine impact (21,22). This design incorporates a sample frame aims for repeatability rather than representativeness, to detect changes in HPV prevalence between successive birth cohorts. The 2 populations are separated by time, and impact is assessed by comparing prevalence of infection in the population with access to the vaccination program, with a reference population that did not have access because they were too old at the time of vaccination program introduction.

### Study procedures

Both surveys followed identical procedures and were administered by trained study staff who participated in routine clinic activities. All adolescent girls aged 17-18 years attending the clinic for health services were invited to join the survey. They were asked to provide written informed consent, but the parental consent requirement was waived following ethics review. Participants completed a computer-assisted self-interview that captured information on sociodemographics, sexual behavior, and HPV vaccination history. Additional medical information on HIV history was collected and abstracted from medical records by study staff. Participants without evidence of an HIV test in the prior 3 months were offered HIV counseling and testing using locally available HIV rapid tests. Participants self-collected vaginal swabs (Copan Diagnostics, Inc, Brescia, Italy) after demonstration by a trained study staff member. Self-collection of samples has previously been demonstrated as feasible and acceptable in this population (23). Dry swabs were shipped on dry ice to a central laboratory and tested using the Seegene Anyplex II HPV28 (Seegene, Seoul, South Korea). WHO proficiency panels were performed at the start of the study, and external quality assurance testing was performed on a subset of specimens at the Royal Women’s Hospital in Australia.

This study was reviewed and approved by the University of the Witwatersrand human research ethics committee (HREC No. 181005) and the University of New South Wales HREC (HREC No. 181-005).

### Statistical considerations

Based on a literature review, we assumed that the prevalence of HPV 16 and 18 in the prevaccine survey would be 19%-30% (24-26). We estimated that we would have 80% power to detect prevalence ratios ranging from 0.4 to 0.8 with a minimum sample of 315 HIV-negative participants and 213 HIV-positive participants in the postvaccine survey. Meta-analyses of vaccine impact data from countries that have implemented 3-dose schedules suggest relative reductions in HPV 16 and 18 prevalence among vaccine-eligible female adolescents (aged 15-19 years) within 1-4 years following program implementation, is 72% if coverage is high (≥50%), and is 50% if coverage is low (<50%), providing a range for sample size estimates given a range of program coverage (4).

Vaccination status was validated against records extracted from national program and single-dose vaccination registers. Probabilistic matching techniques were used to identify previously vaccinated individuals with a probability of 90% or more.

Our primary outcome of interest was vaccine-specific types HPV 16 and 18 prevalence. We restricted comparisons to populations who completed both surveys in the Free State. We used generalized linear models to estimate crude prevalence ratios and 95% confidence intervals (CIs) between the pre-and postvaccination surveys. Models were fitted for prespecified groups of HPV types (see Tables 2, 4, and 6). We adjusted these estimates to account for differences in characteristics across the 2 survey populations. Variables that were associated with each outcome at the 0.10 level in the univariable regression were included in the final adjusted model. We assessed differences in impact by HIV status using stratification. Vaccine effectiveness defined as the direct protective effect of vaccine in individuals vaccinated compared with those unvaccinated in the same population and exposed to the same vaccination program was a secondary outcome and calculated within the postvaccine survey only using the formula (1- adjusted prevalence ratio) x 100 (21).

**Table 1.** Participant characteristics, by survey period

Characteristics	Survey 1 prevaccine survey, No. (%)	Survey 2 postvaccine survey, No. (%)	*P*
	(n = 506)	(n = 892)	
Age, y			
17	252 (49.8)	438 (49.1)	.802
18	254 (50.2)	454 (50.9)	
Currently in school^a^	419 (82.8)	807 (90.5)	<.001
Relationship status			
Single	261 (51.6)	357 (40.0)	<.001
With partner, unmarried	241 (47.6)	533 (59.8)	
Married	4 (0.8)	2 (0.2)	
Cigarette smoking history	55 (10.9)	53 (5.9)	.001
Alcohol use	220 (43.4)	443 (50.0)	<.001
Current contraceptive use	385 (76.1)	531 (59.5)	<.001
Ever had vaginal sex	365 (72.1)	501 (56.2)	<.001
Age at first sex,^b^ y			
14 and younger	18 (5.6)	35 (7.1)	.574
15-16	174 (53.5)	249 (50.8)	
17-18	133 (40.9)	206 (42.1)	
Number of lifetime sex partners^c^			
1	114 (31.2)	192 (38.3)	.088
2	125 (34.3)	148 (29.6)	
≥3	126 (34.5)	161 (32.1)	
Condom use at last sex^c^	188 (51.2)	139 (25.1)	<.001
History of sexually transmitted infection treatment^d^	53 (14.5)	58 (11.5)	<.001
Living with HIV	157 (31.0)	117 (13.1)	<.001

a Missing responses survey 1 (n = 1) and survey 2 (n = 4).

b Denominator is those who ever had vaginal sex; missing responses or preferred not to answer survey 1 (n = 40) and survey 2 (n = 11).

c Denominator is those who ever had vaginal sex.

d Denominator is those who ever had vaginal sex; not captured in survey 1 (n = 141) who never had sex; missing responses or preferred not to answer survey 2 (n = 1).

**Table 2.** Comparison of human papillomavirus (HPV) prevalence by survey period (n = 1398)

HPV type	Crude prevalence		Prevalence ratios		
	Survey 1 prevaccine survey, No. (%; 95% CI)	Survey 2 postvaccine survey, No. (%; 95% CI)	Crude prevalence ratio (95% CI)	Adjusted prevalence ratio (95% CI)^a^	*P* for adjusted prevalence ratio
	(n = 506)	(n = 892)			
HPV 16, 18	117 (23.1; 19.7 to 27.0)	108 (12.1; 10.1 to 14.4)	0.52 (0.41 to 0.66)	0.65 (0.51 to 0.83)	<.001
HPV 16	75 (14.8; 12.0 to 18.2)	65 (7.3; 5.8 to 9.2)	0.49 (0.36 to 0.67)	0.59 (0.43 to 0.82)	.002
HPV 18	56 (11.1; 8.6 to 14.1)	50 (5.6; 4.3 to 7.3)	0.51 (0.35 to 0.73)	0.67 (0.46 to 0.98)	.037
HPV 31, 45	94 (18.6; 15.4 to 22.2)	100 (11.2; 9.3 to 13.5)	0.60 (0.47 to 0.78)	0.74 (0.56 to 0.97)	.030
HPV 33, 35, 39, 51, 52, 56, 58, 59, 68	253 (50.0 45.7 to 54.4)	482; 54.0 (50.8 to 57.3)	1.08 (0.97 to 1.20)	1.14 (1.03 to 1.26)	.011
HPV 33, 52, 58	130 (25.7; 22.1 to 29.7)	206 (23.1; 20.4 to 26.0)	0.90 (0.74 to 1.09)	0.94 (0.78 to 1.14)	.554
HPV 35, 39, 51, 56, 59, 68	207 (40.9; 36.7 to 45.3)	433 (48.5; 45.3 to 51.8)	1.19 (1.05 to 1.34)	1.27 (1.12 to 1.43)	<.001
Any oncogenic HPV excluding 16 and 18	275 (54.4; 50.0 to 58.7)	503 (56.4; 53.1 to 59.6)	1.04 (0.94 to 1.15)	1.10 (1.00 to 1.20)	.043
Any oncogenic HPV	298 (58.9; 54.6 to 63.1)	522 (58.2; 55.3 to 61.7)	0.99 (0.91 to 1.09)	1.03 (0.95 to 1.12)	.444
HPV 6, 11	89 (17.6; 14.5 to 21.2)	170 (19.1; 16.6 to 21.8)	1.08 (0.86 to 1.37)	1.24 (0.98 to 1.58)	.069

a Variables examined for adjusting in the final model: HIV status, being in school, relationship status, smoking, drinking, lifetime number of partners, reported vaginal sex, contraceptive use. CI = confidence interval.

## Results

### Single-dose vaccination campaign

Of 6673 enumerated adolescent girls in grade 10, a total of 4960 returned parental consent forms and were assessed for HPV vaccine eligibility. Of these, 4807 (72%) were eligible and accepted single-dose vaccination. Vaccination coverage by school ranged from 14% to 96% of enumerated learners. The primary reason for nonvaccination in those assessed for vaccine eligibility was lack of parental consent (114 of 153; 75%). The median age of those vaccinated was 16 years (interquartile range [IQR] = 15-17 years).

### Survey participant characteristics

Overall, 585 participants were enrolled in the Free State from June to December 2019. Of these, 77 were excluded because of prior HPV vaccination, while 2 did not have valid HPV DNA results, leaving 506 participants included in the analysis of the prevaccine survey. For the postvaccine survey, 963 participants were enrolled from February to December 2021; 71 were excluded as confirmed vaccine recipients in the national 2-dose program, leaving 892 participants included in the analysis of the postvaccine survey. Most participants were aged 18 years, in school, aged 15 years or older at first sex, and reported 2 or more lifetime sexual partners (Table 1). Participants in the postvaccine survey were significantly more likely to be in school (*P* < .001), to be in a relationship (*P* < .001), and to report alcohol use (*P* < .001) than prevaccine survey participants but were less likely to report a history of cigarette smoking (*P* = .001), current hormonal contraceptive use (*P* < .001), sexual activity (*P* < .001), condom use at last sex (*P* < .001), or a history of STI treatment (*P* < .001). Fewer participants living with HIV were enrolled in the postvaccine survey (*P* < .001). Overall, 20% (175 of 892) of postvaccine survey participants were confirmed as single-dose HPV vaccine recipients. The median interval between single-dose vaccine receipt and survey enrollment was 2.4 years (range = 2.0-2.9 years).

### Impact of single dose on population-level HPV prevalence

The crude prevalence of HPV 16 and 18 was statistically significantly lower in the postvaccine survey (12%, 108 of 892) compared with the prevaccine survey (23%, 117 of 506), with HPV 16 and 18 prevalence estimated to be 35% lower (adjusted prevalence ratio = 0.65, 95% CI = 0.51 to 0.83) in the postvaccine survey after adjusting for differences in the 2 survey populations (Table 2). HPV 16 and 18 prevalence were also significantly lower in the postvaccine survey when examined individually (HPV 16: adjusted prevalence ratio = 0.59, 95% CI = 0.43 to 0.82; HPV 18: adjusted prevalence ratio = 0.67, 95% CI = 0.46 to 0.98). There was some evidence of cross-protection against HPV types 31 and 45, with a 26% relative reduction in HPV 31 and 45 prevalence in the postvaccine survey (adjusted prevalence ratio = 0.74, 95% CI = 0.56 to 0.97). In contrast, there did not appear to be any impact of single-dose HPV vaccination on other oncogenic HPV types 33, 35, 39, 51, 52, 56, 58, 59, and 68 with a higher prevalence observed in the postvaccine survey (adjusted prevalence ratio = 1.14, 95% CI = 1.03 to 1.26). This trend was consistent for any nonvaccine oncogenic HPV types and for low-risk HPV 6 and 11 types that are not included in the bivalent vaccine (Table 2).

### Impact of single dose on population-level HPV prevalence in women living with HIV

In the population living with HIV, postvaccine survey participants were similar to the prevaccine survey but were less likely to report a history of cigarette smoking (*P* = .018), to use hormonal contraception (*P* < .001), to be sexually active (*P* = .009), and to report condom use at last sex (*P* < .001) (Table 3). Postvaccine survey participants living with HIV were more likely to have been diagnosed at a younger age (aged younger than 15 years) (*P* = .004), to have been on antiretroviral therapy (ART) for less than 2 years (*P* = .004), and to have a recent HIV RNA viral load (*P* = .005), although among those with a recent HIV RNA result, viral suppression rates were similar across surveys (*P* = .33).

**Table 3.** Characteristics of participants living with HIV, by survey period

Characteristics	Survey 1 prevaccine survey, No. (%)	Survey 2 postvaccine survey, No. (%)	*P*
	(n = 157)	(n = 117)	
Age, y			
17	68 (43.3)	56 (47.9)	.454
18	89 (56.7)	61 (52.1)	
Currently in school	111 (70.7)	93 (79.5)	.099
Relationship status			
Single	81 (51.6)	52 (44.4)	.242
With partner, unmarried	76 (48.4)	65 (55.6)	
Married	0 (0.0)	0 (0.0)	
Cigarette smoking history	24 (15.3)	6 (5.1)	.018
Alcohol use	69 (43.9)	51 (43.6)	.425
Current contraceptive use	113 (72.0)	54 (46.2)	<.001
Ever had vaginal sex	113 (72.0)	62 (53.0)	.009
Age at first sex,^a^ y			
14 and younger	9 (8.1)	11 (18.0)	.152
15-16	63 (57.3)	30 (49.2)	
17-18	38 (34.6)	20 (32.8)	
Number of lifetime sex partners^b^			
1	32 (28.3)	17 (27.4)	.916
2	39 (34.5)	20 (32.3)	
≥3	42 (37.2)	25 (40.3)	
Condom use at last sex^b^	53 (46.5)	15 (21.4)	<.001
History of sexually transmitted infection treatment^c^	20 (17.9)	11 (15.7)	.501
Age at HIV diagnosis, y			
Younger than 9	34 (21.7)	32 (27.4)	.004
9-14	22 (14.0)	25 (21.4)	
15-18	90 (57.3)	60 (21.3)	
Not available	11 (7.0)	0 (0.0)	
On ART	149 (94.9)	106 (90.6)	.165
Time on ART			.004
Not on ART	8 (5.1)	11 (9.4)	
<2 y	84 (53.5)	91 (77.8)	
≥2 y	64 (40.8)	5 (4.3)	
Not available	1 (0.6)	10 (8.6)	
Most recent HIV RNA viral load			
≤50 log_10_ copies/mL	43 (27.4)	41 (35.0)	.005
>50 log_10_ copies/mL	34 (21.7)	39 (33.3)	
Not available	80 (51.0)	37 (31.6)	

a Denominator is those who ever had vaginal sex; missing responses survey 1 (n = 3) and survey 2 (n = 1). ART = antiretroviral therapy.

b Denominator is those who ever had vaginal sex.

c Denominator is those who ever had vaginal sex; not captured in survey 1 (n = 93) for those who never had sex.

**Figure 1.**
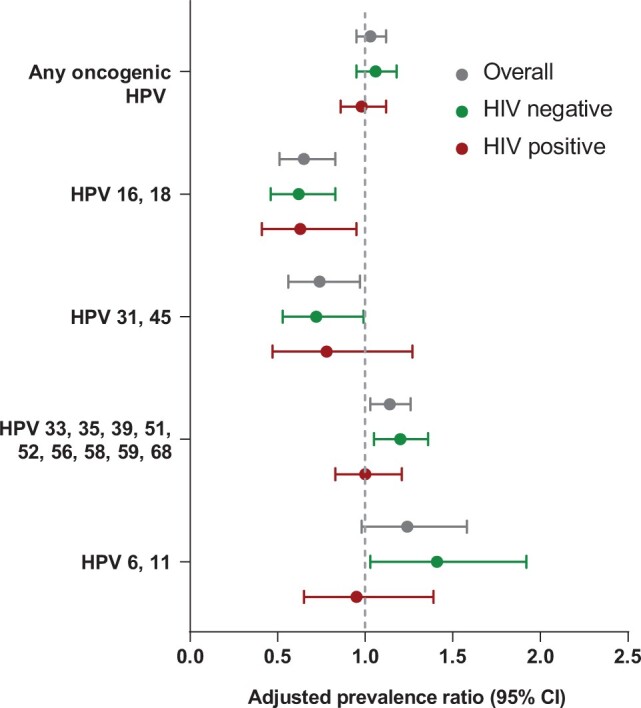
Comparison of human papillomavirus prevalence between prevaccine survey and postvaccine survey, by HIV status. CI = confidence interval; HPV = human papillomavirus.

HPV 16 and 18 prevalence was 37% lower (adjusted prevalence ratio = 0.63, 95% CI = 0.41 to 0.95; *P* = .026) in those living with HIV (20.5%, 24 of 117) in the postvaccine survey compared with the prevaccine survey (33%, 52 of 157), after adjustment (Table 4). Although not statistically significant, there was a trend toward reductions in vaccine-associated types when considered individually (HPV 16: adjusted prevalence ratio = 0.71, 95% CI = 0.40 to 1.24; HPV 18: adjusted prevalence ratio = 0.72, 95% CI = 0.39 to 1.33) or when considering cross-protection types (HPV 31 and 45: adjusted prevalence ratio = 0.78, 95% CI = 0.47 to 1.27). By comparison, single-dose vaccination did not have an impact on the prevalence of other oncogenic HPV types 33, 35, 39, 51, 52, 56, 58, 59, and 68 (adjusted prevalence ratio = CI = 0.83 to 1.21) or nonvaccine low-risk HPV types 6 and 11 (adjusted prevalence ratio = 0.95, 95% CI = 0.65 to 1.39). The overall associations observed in young women living with HIV were consistent with those observed in those not living with HIV (Table 4 and Figure 1).

**Table 4.** Comparison of human papillomavirus (HPV) prevalence by survey period, stratified by HIV status

HPV type	Crude prevalence		Prevalence ratios		
	Survey 1 prevaccine survey, No. (%; 95% CI)	Survey 2 postvaccine survey	Crude prevalence ratio (95% CI)	Adjusted prevalence ratio^a^ (95% CI)	*P* for adjusted prevalence ratio
Living with HIV (n = 274)	(n = 157)	(n =117)			
HPV 16, 18	52 (33.1; 26.2 to 40.9)	24 (20.5; 14.1 to 28.8)	0.62 (0.41 to 0.94)	0.63 (0.41 to 0.95)	.026
HPV 16	29 (18.5; 13.1 to 25.4)	15 (12.8; 7.9 to 20.2)	0.69 (0.39 to 1.23)	0.71 (0.40 to 1.24)	.228
HPV 18	29 (18.5; 13.1 to 25.4)	13 (11.1; 6.6 to 18.2)	0.61 (0.33 to 1.12)	0.72 (0.39 to 1.33)	.291
HPV 31, 45	39 (24.8; 18.7 to 32.2)	18 (15.4; 9.9 to 23.1)	0.62 (0.37 to 1.03)	0.78 (0.47 to 1.27)	.314
HPV 33, 35, 39, 51, 52, 56, 58, 59, 68	97 (61.8; 53.9 to 69.1)	69 (59.0; 49.8 to 67.5)	0.95 (0.79 to 1.16)	1.00 (0.83 to 1.21)	.993
HPV 33, 52, 58	58 (36.9; 29.7 to 44.8)	31 (26.5; 19.3 to 35.3)	0.72 (0.50 to 1.03)	0.78 (0.55 to 1.13)	.189
HPV 35, 39, 51, 56, 59, 68	78 (49.7; 41.9 to 57.5)	64 (54.7; 45.6 to 63.5)	1.10 (0.88 to 1.38)	1.09 (0.88 to 1.36)	.412
Any oncogenic HPV excluding 16, 18	103 (65.6; 57.8 to 72.6)	72 (61.5; 52.4 to 69.9)	0.94 (0.78 to 1.13)	1.02 (0.88 to 1.18)	.799
Any oncogenic HPV	110 (70.1; 62.4 to 76.7)	75 (64.1; 55.0 to 72.3)	0.91 (0.77 to 1.08)	0.98 (0.86 to 1.12)	.802
HPV 6, 11	44 (28.0; 21.5 to 35.6)	31 (26.5; 19.3 to 35.3)	0.95 (0.64 to 1.40)	0.95 (0.65 to 1.39)	.792
Not living with HIV (n = 1124)	(n = 349)	(n = 775)			
HPV 16, 18	65 (18.6; 14.9 to 23.1)	84 (10.8; 8.8 to 13.2)	0.58 (0.43 to 0.78)	0.62 (0.46 to 0.83)	.002
HPV 16	46 (13.2; 10.0 to 17.2)	50 (6.5; 4.9 to 8.4)	0.49 (0.33 to 0.72)	0.54 (0.37 to 0.80)	.002
HPV 18	27 (7.7; 5.4 to 11.1)	37 (4.8; 3.5 to 6.5)	0.62 (0.38 to 1.00)	0.65 (0.40 to 1.06)	.082
HPV 31, 45	55 (15.8; 12.3 to 20.0)	82 (10.6; 8.6 to 13.0)	0.67 (0.49 to 0.92)	0.72 (0.53 to 0.99)	.044
HPV 33, 35, 39, 51, 52, 56, 58, 59, 68	156; 44.7 (39.6 to 50.0)	413 (53.3; 44.1 to 51.1)	1.19 (1.04 to 1.36)	1.20 (1.05 to 1.36)	.007
HPV 33, 52, 58	72 (20.6; 16.7 to 25.2)	175 (22.6; 19.8 to 25.7)	1.09 (0.86 to 1.40)	1.10 (0.86 to 1.40)	.459
HPV 35, 39, 51, 56, 59, 68	129 (37.0; 32.1 to 42.2)	369 (47.6; 44.1 to 51.1)	1.29 (1.10 to 1.51)	1.30 (1.12 to 1.52)	<.001
Any oncogenic HPV excluding 16, 18	172 (49.3; 44.1 to 54.2)	431 (55.6; 52.1 to 59.1)	1.13 (1.00 to 1.28)	1.14 (1.01 to 1.28)	.035
Any oncogenic HPV	188 (53.9; 48.6 to 59.0)	447 (57.7; 54.2 to 61.1)	1.07 (0.96 to 1.20)	1.06 (0.95 to 1.18)	.299
HPV 6, 11	45 (12.9; 9.8 to 16.8)	139 (17.9; 15.4 to 20.8)	1.39 (1.02 to 1.90)	1.41 (1.03 to 1.92)	.033

a HIV negative: adjusted for being in school, relationship status, alcohol use, contraception use, reported vaginal sex, lifetime number of partners. HIV positive: adjusted for lifetime number of partners, all variables retained at *P* ≤ .1 in final adjusted models. CI = confidence interval.

### Single-dose HPV vaccine effectiveness in the postvaccine survey

There were 175 (19.6%) confirmed single-dose recipients in the postvaccine survey (Table 5). Single-dose recipients were slightly older (*P* < .001), more likely to be in school (*P* = .001), and be older at sexual debut (*P* = .001). There was no difference in HIV status between those who did and did not receive single-dose HPV (10% vs 14%, *P* = .137). HPV 16 and 18 prevalence individually or combined was substantially lower in single-dose recipients, reflecting a direct vaccine effectiveness range from 62% to 68% (Table 6). Cross-protection effects against HPV 31 and 45 were also evident (vaccine effectiveness = 53%, 95% CI = 13% to 74%). Protective effects were not observed for any nonvaccine associated HPV types, however (Table 6).

**Table 5.** Comparison of participant characteristics in postvaccine survey, by human papillomavirus (HPV) single-dose vaccination status

Characteristics	Did not receive HPV single-dose vaccine, No. (%) (n = 717)	Received HPV single-dose vaccine, No. (%) (n = 175)	*P*
Age, y			
17	375 (52.3)	63 (36.0)	<.001
18	342 (47.7)	112 (64.0)	
Currently in school^a^	636 (88.7)	171 (97.7)	.001
Relationship status			.139
Single	276 (38.5)	81 (46.3)	
With partner, unmarried	439 (61.2)	94 (53.7)	
Married	2 (0.3)	0 (0.0)	
Cigarette smoking history	45 (6.3)	8 (4.6)	.171
Alcohol use	365 (50.9)	78 (44.6)	.028
Current contraceptive use	423 (59.0)	108 (61.7)	.511
Ever had vaginal sex	402 (56.1)	99 (56.6)	.883
Age at first sex,^b^ y			.001
14 and younger	35 (8.7)	0 (0.0)	
15-16	205 (50.1)	44 (40.7)	
17-18	153 (38.0)	53 (49.1)	
Number of lifetime sex partners^c^			.338
1	161 (40.0)	31 (28.7)	
2	118 (29.3)	30 (27.8)	
≥3	123 (30.1)	38 (35.2)	
Condom use at last sex^c^	150 (37.3)	47 (47.4)	.090
History of sexually transmitted infection treatment^d^	48 (6.7)	10 (10.1)	
Living with HIV	100 (14.0)	17 (9.7)	.137

a Missing responses survey 1 (n = 4).

b Denominator is those who ever had vaginal sex; missing responses survey 1 (n = 9).

c Denominator is those who ever had vaginal sex.

d Denominator is those who ever had vaginal sex; missing responses survey 1(n = 1).

**Table 6.** Comparison of human papillomavirus (HPV) prevalence by HPV single-dose vaccination status and estimated vaccine effectiveness (survey 2 only)

HPV type	Crude prevalence		Prevalence ratios			HPV single-dose vaccine effectiveness (95% CI)
	No HPV single dose received, No. (%; 95% CI)	HPV single dose received, No. (%; 95% CI)	Crude prevalence ratio (95% CI)	Adjusted prevalence ratio^a^ (95% CI)	*P* for adjusted prevalence ratio	
	(n = 717)	(n = 175)				
HPV 16, 18	99 (13.8; 11.5 to 16.5)	9 (5.1; 2.7 to 9.6)	0.37 (0.19 to 0.72)	0.36 (0.19 to 0.70)	.002	64% (30% to 81%)
HPV 16	60 (8.4; 6.6 to 10.6)	5 (2.9; 1.2 to 6.7)	0.34 (0.14 to 0.83)	0.32 (0.13 to 0.78)	.013	68% (22% to 87%)
HPV 18	46 (6.4; 4.8 to 8.5)	4 (2.3; 0.9 to 5.9)	0.36 (0.13 to 0.98	0.38 (0.14 to 1.03)	.058	62% (0% to 86%)
HPV 31, 45	89 (12.4; 10.2 to 15.0)	11 (6.3; 3.5 to 11.0)	0.51 (0.28 to 0.93)	0.47 (0.26 to 0.87)	.016	53% (13% to 74%)
HPV 33, 35, 39, 51, 52, 56, 58, 59, 68	396 (55.2; 51.6 to 58.8)	86 (49.1; 41.8 to 56.2)	0.89 (0.75 to 1.05)	0.90 (0.76 to 1.06)	.195	NS
HPV 33, 52, 58	169 (23.6; 20.6 to 26.8)	37 (21.1; 15.7 to 27.8)	0.90 (0.65 to 1.23)	0.91 (0.66 to 1.24)	.554	NS
HPV 35, 39, 51, 56, 59, 68	354 (49.4; 45.7 to 53.0)	79 (45.1; 37.9 to 52.6)	0.91 (0.76 to 1.06)	0.91 (0.76 to 1.08)	.275	NS
Any oncogenic HPV excluding 16, 18	416 (58.1; 54.4 to 61.6)	87 (49.7; 42.4 to 57.1)	0.86 (0.73 to 1.01)	0.86 (0.74 to 1.01)	.071	NS
Any oncogenic HPV	431 (60.1; 56.5 to 63.6)	91 (52.0; 44.6 to 59.3)	0.87 (0.74 to 1.01)	0.87 (0.75 to 1.01)	.326	NS
HPV 6, 11	133 (18.6; 15.9 to 21.6)	37 (21.1; 15.7 to 27.8)	1.14 (0.82 to 1.58)	1.18 (0.85 to 1.62)	.073	NS

a Adjusted for age, in school, alcohol use, age at first sex, condom use, HIV status. CI = confidence interval; NS = not significant.

A total of 9 single-dose recipients had HPV 16 or 18 detected. Although it is not possible to determine timing of infection precisely relative to vaccination, in 3 of 5 participants for which there is data, age of first sex predates age at vaccination.

## Discussion

In this population impact study using repeat cross-sectional surveys in adolescent girls aged 17-18 years attending public sector primary health-care services, we observed declines in the prevalence of both vaccine-specific HPV types 16 and 18 and cross-protection HPV types 31 and 45 two years after a single-dose HPV vaccine campaign with the bivalent vaccine in a district in the Free State, South Africa. By contrast, we did not observe changes in the prevalence of nonvaccine-associated HPV types. The latter finding provides supportive evidence that this is a true biological effect and not the result of differences in sexual behavior between the 2 surveys. The lower prevalence of vaccine-specific HPV types in the postvaccine survey despite evidence of higher risk behaviors suggests both direct and herd protection effects following an overall reduction in circulating vaccine-specific HPV types after the single-dose campaign in 2019. These results are consistent with trends from similar population-impact studies in high-income country settings and, to our knowledge, are the first evidence of population impact of single-dose HPV vaccination on HPV 16 and 18 in an LMIC setting (27).

The population impact of single-dose HPV vaccination did not differ by HIV status, with similar reductions observed in young women with HIV and without HIV. We hypothesize that HIV acquisition was recent and the result of sexual activity during adolescence given that the majority had been on ART for less than 2 years. The high proportion on ART reflects the clinic population from which they were drawn. Substantial efforts have been made to increase testing and linkage to care among adolescents in this district through PEPFAR support over the study period (28). It may be that less favorable impacts are seen in populations or settings where ART coverage is low, given the effects of HIV immune suppression in promoting HPV persistence and oncogenesis, as well as the potential for lower vaccine efficacy in people living with HIV (29). Although HPV vaccine efficacy data in people living with HIV are limited, studies of immunogenicity in response to multiple vaccine doses suggest that individuals with high CD4+ count or on ART have robust immune responses to vaccine that are preserved 2-4 years. More direct evidence is needed however to confirm the efficacy against vaccine-specific HPV infection in people living with HIV, particularly for single dose (14). Nevertheless, these data provide some reassurance for high HIV burden countries considering adoption of the WHO recommendation on single-dose vaccination.

Although the overall vaccine impact was modest compared with other settings, this may be the result of low population vaccination coverage with only 20% of those in the postvaccine survey being single-dose recipients (despite 72% of grade 10 girls accepting a single dose during the campaign in 2019) and the short interval between vaccination and postvaccine survey. Possible explanations include the wide age range of girls in grade 10 vaccinated in 2019, which included 25% already aged 17 years and older and therefore too old for inclusion in the postvaccine survey in 2021, the presence at public sector clinics of girls who were already out of school in 2019 and therefore would not have received the single dose, out migration of single-dose recipients from the district, or the effects of COVID-19 on health-seeking behavior. Despite the potential influence of these factors, we observed an impact on population HPV 16 and 18 prevalence. Data from modeling studies suggest strong population-level impacts can be expected with coverage as low as 20% (30).

We observed a higher crude prevalence of nonvaccine HPV types in the postvaccine survey. The most plausible explanation is that this is due to higher sexual risk behavior in the postvaccine survey because of the COVID-19 pandemic. Fewer participants reported previous STI treatment or contraceptive use, and condom use at last sex was less frequent, reflecting decreased access to preventive and curative health-care services during the pandemic. The South African HIV program demonstrated remarkable resilience in the face of COVID-19 disruptions overall, however, reports suggest that adolescents fared worse in terms of access to HIV and sexual and reproductive health care, as well as access to social and educational support structures that may be protective (31,32). HPV-type replacement is unlikely given the genetic stability of HPV, with analyses from clinical cohorts suggesting a low probability of this, although it may be too early to confirm this; ongoing surveillance of nonvaccine types remains critical (33-35).

There are several limitations to this study. HPV prevalence estimates may not be generalizable to all South African women given the clinic-based design. However, this design does not aim to be representative but rather to be reproducible and to demonstrate relative reductions in prevalence over time in similarly recruited populations. By focusing on an age group that is sexually active, we increased the likelihood of detecting HPV infection and changes in infection prevalence in successive postvaccination age cohorts. There were differences in sexual behavior between the pre- and postvaccine survey. Although we did not see any impact on the prevalence of nonvaccine HPV types, and we adjusted for differences in sexual behavior between the 2 surveys, we cannot exclude the possibility of incomplete adjustment or further confounding by unmeasured factors. Any bias introduced through higher risk behavior however would likely diminish the apparent effectiveness that we observed. We were unable to assess the proportion of participants who had already been exposed to HPV 16 and 18 at the time of single-dose vaccination because we did not collect behavioral or infection information during the campaign. Exposure to HPV 16 and 18 prior to vaccination may have diminished estimates of direct vaccine effectiveness in the postvaccine survey, and there is some evidence to support this. We did not investigate single-dose vaccine effectiveness by HIV status. Rather, we evaluated single-dose vaccine program impact by HIV status. More research is needed to evaluate single-dose effectiveness among people living with HIV.

Despite these limitations, our study has several strengths. The pre- and postvaccination campaign repeat cross-sectional survey design has been recommended for impact assessment of vaccination programs (21). Our sample collection, transport, and testing procedures were identical across the 2 surveys. Any deterioration of sensitivity due to transportation, storage, or other factors would therefore be nondifferential in regard to the survey round and HPV type. The observed reduction in detection of HPV 16 and 18 but not the nonvaccine HPV types between the pre- and postvaccine survey cohorts was consistent with our hypothesis of vaccine impact and highly unlikely to be an artefact of changes in specimen quality. We were able to verify vaccination status based on vaccination registers held by the national program and through our own single-dose campaign. The clinic-based sampling strategy was efficient and allowed us to standardize recruitment over repeated time points and demonstrate population impact of single-dose HPV vaccination within programmatic time frames. This sentinel surveillance approach can now be used for ongoing monitoring of the South African HPV vaccination program.

In summary, we confirmed a decrease in the population prevalence of vaccine-specific and cross-protection HPV types 2 years after a single-dose HPV vaccine campaign in a single district in South Africa. These results were consistent irrespective of HIV status and provide reassurance to countries in eastern and southern Africa contemplating a shift to single-dose HPV vaccination. Single-dose HPV vaccination has the potential to increase HPV vaccination coverage rates in regions where the burden of cervical cancer is high and where HPV vaccination introduction and coverage have been low.

## Data Availability

Data collected for this study may be made available on request. On completion of the study, data will be shared through a general data sharing repository, figshare.com, in accordance with University of the Witwatersrand data sharing policies.
